# Baseline assessment of staff perception of critical value practices in government hospitals in Kuwait

**DOI:** 10.1186/s12913-022-08329-z

**Published:** 2022-08-03

**Authors:** Talal ALFadhalah, Buthaina Al Mudaf, Haya Al Tawalah, Wadha A. Al Fouzan, Gheed Al Salem, Hanaa A. Alghanim, Samaa Zenhom Ibrahim, Hossam Elamir, Hamad Al Kharji

**Affiliations:** 1 Quality and Accreditation Directorate, Ministry of Health, Kuwait City, Kuwait; 2 Assistant Undersecretary of Public Health Affairs, Ministry of Health, Kuwait City, Kuwait; 3 Laboratory Department, Ministry of Health, Yacoub Behbehani Center, Sulaibikhat, Kuwait; 4 Microbiology Department, Faculty of Medicine, Kuwait University, Jabriya, Kuwait; 5 Laboratory Department, Farwania Hospital, Ministry of Health, Farwania, Kuwait; 6 Accreditation Affairs Department, Quality and Accreditation Directorate, Ministry of Health, Kuwait City, Kuwait; 7 Safety Department, Quality and Accreditation Directorate, Ministry of Health, Kuwait City, Kuwait; 8 Department of Health Management, Planning and Policy, High Institute of Public Health, Alexandria University, Alexandria, Egypt; 9 Research and Technical Support Department, Quality and Accreditation Directorate, Ministry of Health, Kuwait City, Kuwait

**Keywords:** Kuwait, Hospital Laboratory, Critical values reporting, Hospital practices

## Abstract

**Background:**

Notification of laboratory-determined critical values is key for effective clinical decision making and is thus a consequential step in a patient’s health care and safety. This study presents an overview of staff reporting policies and procedures concerning critical values in Kuwaiti governmental hospitals.

**Methods:**

A cross-sectional descriptive study design was adopted. Study subjects were affiliated with laboratories from five government hospitals (four general and one sub-specialty hospital). All laboratory staff in every hospital were included. The Statistical Package for the Social Sciences (version 23) was used to analyse the collected data at a significance level of ≤ 0.05. Quantitative data analysis included univariate descriptive (means, medians, standard deviations, frequencies, percentages) and bivariate (chi-squared, ANOVA and Kruskal–Wallis tests) analyses. These analyses provided associations between participating hospitals and staff perceptions towards the policies and procedures surrounding critical values.

**Results:**

559 questionnaires were returned, a total response of 30.5% after those of 79 phlebotomists were excluded (eligible sample size *n* = 1833). The notification of critical values differs between participated laboratories in delivering protocol and time duration. Linked protocols between laboratories did not exist regarding policies and guidelines for applying the same procedures for critical value notification. There are differences in critical value limits among the participating laboratories.

**Conclusion:**

This study is the first to survey laboratory staff perceptions of critical value practices in Kuwaiti government hospitals. Enhancing critical value reporting and policy is crucial for improving patient safety and to develop high-quality health services. The findings of this study can help policy makers implement future intervention studies to enhance laboratory practices in the area of critical values and improve patient safety and the quality of government hospital systems.

**Supplementary Information:**

The online version contains supplementary material available at 10.1186/s12913-022-08329-z.

## Background

Laboratory tests are estimated to influence 70% of medical diagnoses, which is important to acknowledge, as medical errors based on the results of these tests affect patient safety [1]. The "critical values" yielded by these tests are considered important parameters for evaluating patient safety and clinical efficacy [2]. In a seminal paper published 50 years ago, Lundberg first described the concept of the critical value (also known as a "panic value") as a test result significantly outside the normal range that requires immediate action to be taken by caregiving staff to avoid life-threatening consequences [3]. Since then, the idea of critical laboratory values has prevailed in most medical settings. McFarlane et al. [4] recommended that each laboratory should have their own list of critical values and must report when a result falls outside the determined limits. Regrettably, issues of timely notification and not following-up a critical value persist within present health care systems [4, 5]. Furthermore, critical laboratory values are yet to be standardised throughout health care systems, which requires individual health care organisations to decide their own [5, 6]. In 2004, the World Health Organization stated that the reporting of critical laboratory values is a key goal towards patient safety and it is also an important element in many worldwide accreditation programmes [4, 5]. For example, instant notification of a critical laboratory value is required of medical laboratories abiding by ISO 15,189 guidelines [7]. Moreover, the reporting of critical values usually follows strict criteria to avoid complications in doing [2] so; for example, to simplify the notification process, the College of American Pathologists has guidance on policies and procedures for identifying and reporting critical values [8]. Thus far, the process of reporting critical laboratory values is unknown and no documentation is saved or exchanged between the laboratory and the physician in charge [9]. Furthermore, the volume of forgotten call backs from physicians is considered the major challenge confronting laboratory technicians [8]. In Kuwait, it is unknown how the notification of critical laboratory values, the limits of these values and the policies or guidelines that govern their notification are perceived by hospital staff. The current study aimed to assess these perceptions and practices. In particular, it examined staff knowledge about the availability of policies and how to implement them in a notification scenario, and their knowledge regarding critical values limits. The findings of this study should guide policies aimed at improving patient-centred laboratory practices and governmental procedures. Also, the conclusions of this study might lead to increased patient safety and especially a decrease in the number of lethal mistakes arising from practicing the notification of critical values.

### Stating the problem

Failures to notify on critical laboratory values affect patient safety, and is considered a medical error that could lead to a life-threatening condition [7]. Having policies and guidelines that instruct how and when to notify of a critical value, is a key element that steers the notification process [8]. The current study investigated the knowledge of laboratory staff about such guidelines and policies in Kuwaiti hospitals.

### Significance of research

The current study builds knowledge on the importance to patient safety of the process of critical value notification. Also, by following the policies and guidelines relating to critical value notification, the quality of services will improve services quality, which is expected to lead to a reduction in medical errors.

### Research objectives

The broader objectives were to assess staff perceptions about the notification of critical values. Specifically, we aimed to:

1. Assess the knowledge and attitudes of respondents in government hospitals towards the policies and guidelines relating to the notification of critical values.
2. Assess the respondents knowledge of critical value limits.

## Methods

### Study design

A cross-sectional descriptive study design was adopted. The sample population was laboratory staff in the selected hospitals who are eligible for the study.

### Study settings and study period

Subjects were affiliated with laboratories from five government hospitals. The study areas comprised four general hospitals, providing predominantly secondary care services, and one sub-speciality hospital, which provides tertiary care. The data took around four months to be gathered.

### Research tool

A structured, validated questionnaire was adapted from a published study by Mosallam and Ibrahim [5]. Their questionnaire was divided into seven sections including socio-demographics. The first recorded the characteristics of the partaking hospitals and laboratories. The second section assessed their policies and procedures for reporting critical values, and the third asked about reporting processes. The fourth discussed the way of critical valued documentation and monitoring. The fifth section asked respondents to record ranges of critical values for selected common laboratory tests. Prior final section was asking about any delay in critical values reporting and the reason if that delay applies. The final part of the questionnaire assessed the satisfaction for the critical values reporting process.

The validity of that study tool was assessed by laboratory-setting experts. In our version, the section on socio-demographic characteristics has been modified due to some of the job titles differing. The survey consists of 35 items grouped into seven sections including socio-demographics. The first section focused on the socio-demographic characteristics of the participants. The second section was concerned with the policies and procedures surrounding critical values at participating laboratories. The third section surveyed participants’ practices in reporting critical values to determine, for example, if critical values are communicated immediately upon their identification; also, whether a laboratory technologist or doctor is responsible for actioning the reporting of a critical value if one arises. The fourth section was about how critical values are documented and monitored. The fifth section dealt with critical values for commonly performed tests at the participating hospitals. The sixth section concerned delays in reporting critical values and the reasons for such delays. The final section sought to determine if the participant is satisfied with the process of reporting critical values and if the reporting is subject to delays. Hard copies of the survey in either Arabic (Additional file: Appendix 1) or English (Additional file: Appendix 2) were deployed in the study according to the participant's preference.

### Data collection and sampling

A total population sampling technique was used, and a total of 1833 doctors and technicians from the laboratory departments of the five participating Kuwaiti government hospitals were eligible to participate. Same-day permission was obtained from the laboratory head to enter and distribute the questionnaire. This sampling technique involves the entire population (laboratory staff), so we excluded staff whose duties are not relevant to analysis or reporting (phlebotomists) as well as administration staff. The sampling procedure was based on non-probability. The independent variables were policy and guidelines on critical value notification, whereas the dependent variables were knowledge of the practices and critical value limits. The instruments of data collection were the survey forms distributed for completion by each participant at participating hospitals. Data was gathered separately from each hospital and took four months to complete the collection (November 2018 to February 2019).

### Operational definition

Definition of variables:

**Table:** 

**Policy and guidelines**	Outlines of what to do in any situation involving a laboratory
Knowledge	Information about specific practices
Limits	Reference ranges for critical values (high and low)
Doctor	Laboratory physician
Technician	Laboratory staff (non-physician)
Phlebotomist	Staff trained in withdrawing blood from patients
Critical values	Abnormal limits in laboratory tests ranges

### Data analysis and processing

Participant identity and hospitals were coded for anonymity. To be included in the analysis, a returned questionnaire must have had at least one question answered. The Statistical Package for the Social Sciences (SPSS) version 23 was used to analyse the collected data at a significance level of ≤ 0.05. Quantitative data analysis included univariate descriptive (means, medians, standard deviations, frequencies, percentages) and bivariate (chi-square, ANOVA and Kruskal–Wallis tests) analyses.

## Results

Five hundred and fifty-nine questionnaires were returned from the five participating hospitals, a total response of 30.5% after those of 79 phlebotomists were excluded (eligible sample size *n* = 1833).

### Hospital statistics and socio-demographic characteristics

The number of beds in the hospitals ranged from 218 to 868, with a total number of 2990 and occupancy rates between 54 and 70%. The total number of laboratory units was 38, with each hospital having between 5 and 14 units of different specialities (e.g., microbiology, biochemistry). Collectively, the hospitals performed a total of more than 25 million tests; individually, the numbers of tests ranged from 1.684 million to 7.441 million. The response rate was 30.5%, just under two-thirds of which were from female participants. More than half of the participants were aged between 30 and 45 years and two-thirds of participants were non-Kuwaiti nationals. Doctors comprised 12% of the respondents. Respondents principally worked in haematology (32.8%), biochemistry (23%) and microbiology (20.2%) units. A comparison of hospitals by laboratory position (e.g., head of unit, senior technician) yielded a *p* value of 0.01, that is, they differed significantly in terms of the positions held by their participants. Table 1 shows hospital data and socio-demographic characteristics of the participants.

**Table 1:** Hospital statistics (2018) and socio-demographic characteristics of participants

	Hospital A		Hospital B		Hospital C		Hospital D		Hospital E		*p*	Total	
	N	%	N	%	N	%	N	%	N	%		N	%
Hospital													
Bed number	218	(7.3)	414	(13.8)	868	(29.0)	725	(24.2)	765	(25.6)		2990	
Bed occupancy rate	54%		70%		59%		66%		69%			64.4%	
Laboratories													
Unit number	14	(36.8)	6	(15.8)	5	(13.2)	8	(21.1)	5	(13.2)		38	
Staff number													
Doctors	33	(19.5)	26	(12.6)	26	(8.8)	60	(5.9)	26	(11.4)		171	(8.9)
Technicians	121	(71.6)	169	(81.6)	242	(82.0)	941	(93.0)	189	(82.5)		1662	(86.9)
Phlebotomists	15	(8.9)	12	(5.8)	27	(9.2)	11	(1.1)	14	(6.1)		79	(4.1)
Test number (in millions)	1.684	(6.6)	7.441	(29.0)	4.518	(17.6)	6.343	(24.7)	5.698	(22.2)		25.684	
Participants													
Response rate	42.2%		46.7%		58.2%		10.0%		68.8%			30.6%	
Gender											< .001		
Male	23	(35.4)	23	(25.3)	69	(44.5)	28	(28.0)	73	(49.3)		216	(38.6)
Female	42	(64.6)	68	(74.7)	86	(55.5)	72	(72.0)	75	(50.7)		343	(61.4)
Age											.166		
Below 30 years	27	(41.5)	31	(34.1)	34	(21.8)	31	(31.6)	53	(35.8)		176	(31.5)
30–45 years	31	(47.7)	41	(45.1)	90	(57.7)	53	(54.1)	71	(48.0)		286	(51.3)
46–55 years	5	(7.7)	9	(9.9)	18	(11.5)	7	(7.1)	16	(10.8)		55	(9.9)
Over 55 years	2	(3.1)	10	(11.0)	14	(9.0)	7	(7.1)	8	(5.4)		41	(7.3)
Nationality											< .001		
Kuwaiti	36	(55.4)	47	(51.6)	46	(29.7)	52	(52.0)	17	(11.6)		198	(35.5)
Non-Kuwaiti	29	(44.6)	44	(48.4)	109	(70.3)	48	(48.0)	130	(88.4)		360	(64.5)
Profession											.002		
* Doctor*	*10*	*(15.4)*	*14*	*(15.6)*	*5*	*(3.2)*	*18*	*(18.0)*	*20*	*(13.6)*	.378	*67*	*(12.0)*
Assistant register	−	−	1	(7.1)	−	−	5	(27.8)	1	(5.0)		7	(10.4)
Register	3	(30.0)	3	(21.4)	2	(40.0)	5	(27.8)	11	(55.0)		24	(35.8)
Senior register	3	(30.0)	3	(21.4)	3	(60.0)	3	(16.7)	3	(15.0)		15	(22.4)
Specialist	1	(10.0)	3	(21.4)	−	−	3	(16.7)	2	(10.0)		9	(13.4)
Senior specialist	1	(10.0)	1	(7.1)	−	−	1	(5.6)	2	(10.0)		5	(7.5)
Consultant	2	(20.0)	3	(21.4)	−	−	1	(5.6)	1	(5.0)		7	(10.4)
* Technician*	*55*	*(84.6)*	*76*	*(84.4)*	*151*	*(96.8)*	*82*	*(82.0)*	*127*	*(86.4)*	< .001	*491*	*(88.0)*
Assistant practitioner	2	(3.6)	5	(6.6)	7	(4.7)	6	(7.4)	8	(6.3)		28	(5.7)
Practitioner	13	(23.6)	6	(7.9)	5	(3.3)	9	(11.1)	2	(1.6)		35	(7.2)
Senior practitioner	5	(9.1)	−	−	8	(5.3)	4	(4.9)	4	(3.1)		21	(4.3)
Assistant technician	−	−	3	(3.9)	10	(6.7)	1	(1.2)	8	(6.3)		22	(4.5)
Technician	28	(50.9)	37	(48.7)	71	(47.3)	42	(51.9)	85	(66.9)		263	(53.8)
Senior technician	4	(7.3)	7	(9.2)	21	(14.0)	3	(3.7)	4	(3.1)		39	(8.0)
Specialist	3	(5.5)	9	(11.8)	10	(6.7)	6	(7.4)	7	(5.5)		35	(7.2)
Senior specialist	−	−	9	(11.8)	18	(12.0)	10	(12.3)	9	(7.1)		46	(9.4)
Position											.010		
Head of department	3	(4.8)	1	(1.1)	1	(0.7)	−	−	−	−		5	(0.9)
Head of unit	2	(3.2)	3	(3.4)	2	(1.3)	−	−	4	(2.8)		11	(2.0)
Laboratory doctor	6	(9.5)	10	(11.2)	4	(2.7)	15	(15.6)	16	(11.1)		51	(9.4)
Head of laboratory technicians	2	(3.2)	4	(4.5)	6	(4.0)	3	(3.1)	2	(1.4)		17	(3.1)
Technician	50	(79.4)	71	(79.8)	137	(91.3)	78	(81.3)	122	(84.7)		458	(84.5)
Unit											< .001		
Microbiology	−	−	19	(20.9)	35	(24.1)	20	(20.0)	37	(25.0)		111	(20.2)
Hematology	1	(1.5)	31	(34.1)	50	(34.5)	42	(42.0)	56	(37.8)		180	(32.8)
Biochemistry	11	(16.9)	27	(29.7)	44	(30.3)	1	(1.0)	43	(29.1)		126	(23.0)
Histopathology	13	(20.0)	6	(6.6)	10	(6.9)	17	(17.0)	5	(3.4)		51	(9.3)
Immunology	16	(24.6)	−	−	−	−	11	(11.0)	−	−		27	(4.9)
Virology	15	(23.1)	−	−	6	(4.1)	9	(9.0)	7	(4.7)		37	(6.7)
Molecular genetics	9	(13.8)	−	−		−	−	−	−	−		9	(1.6)
Reception	−	−	8	(8.8)		−	−	−	−	−		8	(1.5)

*N*: Valid responses %: Percentage *p*: *p*-value (Statistically significant at *p* ≤ .05, highly significant at *p* ≤ .001

### Critical value policies and procedures

In completing the statement "Critical values reporting is…", 88.4% of respondents viewed it as "an essential procedure", 7.6% selected "somewhat important" and 4.1% thought it a "minor policy". The hospital best representing that view is hospital D at 7.2% (the most for minor) and hospital E (the most for essential) at 93.2%. Across the five hospitals, 89.5% confirmed the presence of a written procedure for the reporting of critical values, and 86.6% stated that a comprehensive list of critical values exists in their setting. The number of tests in those lists ranged from 0 to 90, with a mean of 12.67 and median of 10, but were not significantly different between the hospitals. Across the five hospitals, 32.1% of participants stated that critical value list was developed based on “medical society recommendations”. Overall, this was the most popular response, but not representative of every hospital, from which differences in response were highly significant. However, a total of 69 individuals (12.3%) gave no reason for how the critical value list was developed; at the single-hospital level, this number varied between 1 (hospital E) and 21 (hospital C). The percentage of laboratory personnel trained in reporting critical values differed significantly between hospitals but was nonetheless high; overall the proportion was greater than 92%. Furthermore, the hospitals were shown to be highly significantly different in their updating of procedures related to critical values; overall, 85.5% of respondents said their laboratory did so. Overall, 63.6% and 41.4% of respondents confirmed their laboratories held “unique ranges for distinct populations" by age and diagnosis, respectively; both sets of responses differed highly significantly between hospitals (*p* < 0.001). The lowest percentage response confirming unique ranges for distinct populations by diagnosis was from hospital B (32.5%) and the highest goes to hospital E at 47.9% according to the responses. Overall, 84.4% of respondents indicated their laboratory had a policy for assessing the timeliness of reporting and differences between hospitals were highly significant. Across all settings, 87.9% of respondents indicated their laboratory had a policy for managing the repetition of critical values and differences between hospitals were significant. The existence of a read-back policy for reported critical values was indicated by 78.8% of all respondents and differences between hospitals were highly significant; hospital B gave the highest percentage (92%) of positive responses to this question. Table 2 shows the responses on policies and procedures pertaining to critical values.

**Table 2:** Critical value policies and procedures at participating laboratories

	Hospital A		Hospital B		Hospital C		Hospital D		Hospital E		*p*	Total	
	*n*	(%)	*n*	(%)	*n*	(%)	*n*	(%)	*n*	(%)		*n*	(%)
Critical values reporting is											.026		
Minor policy	2	(3.3)	1	(1.1)	7	(4.6)	7	(7.2)	5	(3.4)		22	(4.1)
Somewhat important	8	(13.3)	9	(10.2)	16	(10.5)	3	(3.1)	5	(3.4)		41	(7.6)
An essential procedure	50	(83.3)	78	(88.6)	129	(84.9)	87	(89.7)	136	(93.2)		480	(88.4)
Presence of written procedures for critical value reporting	53	(82.8)	80	(89.9)	138	(90.2)	83	(83)	142	(95.9)	.011	496	(89.5)
Presence of a comprehensive list of critical values	41	(69.5)	76	(84.4)	137	(89.0)	74	(79.6)	142	(96.6)	< .001	470	(86.6)
Number of tests in the list													
Range	0–32		4–50		1–30		2–90		3–25			0–90	
Mean ± SD	13.92 ± 10.26		15.02 ± 7.06		12.28 ± 7.34		12.02 ± 16.50		11.76 ± 7.20		.237	12.67 ± 9.36	
Median	15.5		16		10		5		9			10	
Critical value list was developed based on^†^													
Published literature	17	(26.2)	26	(28.6)	26	(16.7)	21	(21.0)	26	(17.6)	.134	116	(20.7)
Opinion of clinicians	10	(15.4)	32	(35.2)	14	(9.0)	30	(30.0)	47	(31.0)	< .001	133	(23.2)
Medical societies' recommendations	9	(13.8)	37	(40.7)	35	(22.4)	30	(30.0)	69	(46.0)	< .001	180	(32.1)
Manufacturer's recommendations	3	(4.6)	9	(9.9)	14	(9.0)	16	(16.0)	10	(6.8)	.084	52	(9.3)
Review of laboratory practice	20	(30.8)	38	(41.8)	54	(34.6)	21	(21.0)	32	(21.6)	.002	165	(29.5)
Informal laboratory peer review	6	(9.2)	10	(11.0)	3	(1.9)	4	(4.0)	2	(1.4)	.001	25	(4.5)
Others	7	(10.8)	2	(2.2)	10	(6.4)	1	(1.0)	11	(7.4)	.032	31	(5.5)
No answers	16	(24.6)	13	(14.3)	21	(13.5)	18	(18.0)	1	(0.7)	< .001	69	(12.3)
Laboratory personnel trained on reporting critical values	52	(83.9)	85	(94.4)	144	(93.5)	87	(88.8)	140	(95.2)	.046	508	(92.2)
Critical values procedures are regularly updated	49	(79.0)	78	(89.7)	118	(77.6)	79	(82.3)	141	(95.9)	< .001	465	(85.5)
Unique ranges for distinct population by age	14	(22.2)	66	(73.3)	111	(72.5)	52	(54.2)	105	(72.4)	< .001	348	(63.6)
Unique ranges for distinct population by diagnosis	23	(35.4)	26	(32.5)	59	(40.4)	44	(44.9)	68	(47.9)	.001	220	(41.4)
Policy for assessing timeliness of reporting	59	(90.8)	70	(79.5)	113	(74.3)	87	(88.8)	135	(91.8)	< .001	464	(84.4)
Policy on how to manage the repetition of critical values	51	(79.7)	75	(90.4)	136	(87.7)	82	(84.5)	135	(92.5)	.002	479	(87.9)
Laboratory policy requires read back	20	(35.7)	81	(92.0)	123	(80.4)	72	(75.0)	127	(88.2)	< .001	423	(78.8)

*N* Number, *%* Percentage, *SD* Standard deviation

^†^ Multiple responses were allowed

*p*: *p* value (statistically significant if *p* ≤ .05, highly significant if *p* ≤ .001)

### Participants’ practices and reporting

The vast majority of participants (91.6%) across all the hospitals reported that wholly critical values are communicated immediately as they arise. In most of the hospitals, the consensus was that a laboratory technician is responsible for alerting the relevant caregiver when a patient presents a critical value, although there was a highly significant difference (*p* < 0.001) between them. Overall, 62.9% of the total number of participants agreed. According to the overall response, the physician ordering the test (63.7%) and nurses (60.5%) are those most likely to receive the calls. Moreover, critical value reporting in the surveyed hospitals showed highly statistically significant differences with respect to physician ordering the test, nurses and any physician on call. Critical values are reported to the caregiver mainly by telephone, as the majority (65.7%) of respondents indicated. Hospitals showed statistically significant differences (*p* = 0.032) in their use of wireless technologies to report critical values; overall, 33.1% of participants indicated this was in use in their setting. A high percentage (91.3%) of respondents across the hospitals reported the re-testing of critical values before verification, but with highly significant differences (*p* < 0.001) between the hospitals. For sample re-testing, the person who is responsible for drawing the sample is contacted to verify the validity of it. A total of 58.4% of respondents confirmed this was procedure at their setting, and there were statistically highly significant differences between the hospitals. The usual practice in handling repeated critical values from the same patient is to report initial critical value and every subsequent critical value, as confirmed by 44.6% of responses, with high significant differences between hospitals. Approximately half (51.2%) of respondents indicated that critical laboratory values are documented in a computer system, written on the result form and documented in the laboratory register. Across all hospitals, 49.8% of surveyed staff stated that when documenting a verbal communication on a log, all information was included (e.g., patient identity, sender identity). The percentage of participants indicating that their setting measured the time from the result becoming available to notification of the caregiver responsible varied significantly between hospitals; across all settings, the percentage was 72.2%. There are many reasons for delaying the reporting of critical values, but approximately 42.5% of respondents across all hospitals claimed that the main issue is the health care provider's contact information being unavailable. Of all the hospital staff surveyed, 86.8% were satisfied with the way critical laboratory values are reported, although the differences between hospitals were statistically significant. The average time for a responsible caregiver to be notified with a test result can differ depending on the shift. In the morning shift, this time ranges between 0 and 4320 min. In the evening, the range is 0 to 2880 min and in the night shift the range is 0 to 1440 min. Differences in notification time during the morning and evening shifts were statistically significant between the hospitals. Table 3 shows the surveyed data regarding critical value reporting practices (participants were allowed to give multiple responses to some of the questions).

**Table 3:** Participants’ Practices and Attitude Towards Critical Value Reporting

	Hospital A		Hospital B		Hospital C		Hospital D		Hospital E		*p*	Total	
	n	(%)	n	(%)	n	(%)	n	(%)	n	(%)		n	(%)
Communicate critical values immediately upon their identification	51	(83.6)	80	(92.0)	144	(93.5)	86	(86.9)	140	(95.9)	.036	501	(91.6)
Person responsible for making the call^†^													
Senior staff	15	(23.1)	54	(59.3)	73	(46.8)	43	(43.0)	79	(53.4)	< .001	264	(47.1)
Laboratory technician	16	(24.6)	70	(76.9)	97	(62.2)	67	(67.0)	102	(68.9)	< .001	352	(62.9)
Others	41	(63.1)	19	(20.9)	22	(14.1)	24	(24.0)	32	(21.6)	< .001	138	(24.6)
No answers	5	(7.7)	3	(3.3)	3	(1.9)	4	(4.0)	1	(0.7)	.053	16	(2.9)
Person who receives the call^†^													
Physicians ordering the test	42	(64.6)	57	(62.6)	92	(59.0)	52	(52.0)	114	(77.0)	.001	357	(63.7)
Nurses	25	(38.5)	72	(79.1)	73	(46.8)	59	(59.0)	110	(74.3)	< .001	339	(60.5)
Any physician on call	28	(43.1)	42	(46.2)	40	(25.6)	39	(39.0)	82	(55.4)	< .001	231	(41.3)
Any people working on the ward	7	(10.8)	11	(12.1)	14	(9.0)	15	(15.0)	9	(6.1)	.201	56	(10.0)
Others	4	(6.2)	6	(6.6)	7	(4.5)	2	(2.0)	3	(2.0)	.252	22	(3.9)
No answers	5	(7.7)	1	(1.1)	3	(1.9)	5	(5.0)	1	(0.7)	.021	15	(2.7)
Critical values are reported to the caregiver mainly by^†^													
Sending test report to ward	28	(43.1)	36	(39.6)	36	(23.1)	33	(33.0)	22	(14.9)	< .001	155	(27.7)
Telephone	40	(61.5)	57	(62.6)	95	(60.9)	55	(55.0)	121	(81.8)	< .001	368	(65.7)
Computer	11	(16.9)	34	(37.4)	49	(31.4)	54	(54.0)	57	(38.5)	< .001	205	(36.6)
Direct contact with requesting physician	34	(52.3)	20	(22.0)	74	(47.4)	33	(33.0)	34	(23.0)	< .001	195	(34.8)
Fax	0	(0.0)	0	(0.0)	1	(0.6)	1	(1.0)	0	(0.0)	.855	2	(0.4)
All tools	10	(15.4)	28	(30.8)	15	(9.6)	24	(24.0)	14	(9.5)	< .001	91	(16.3)
No answers	8	(12.3)	1	(1.1)	2	(1.3)	1	(1.0)	1	(0.7)	< .001	13	(2.3)
Wireless technologies used to report critical values	18	(29.5)	31	(35.2)	52	(34.2)	42	(43.8)	36	(25.0)	.032	179	(33.1)
Re-testing for critical values verification	49	(80.3)	81	(92.0)	152	(97.4)	79	(79.8)	141	(96.6)	< .001	502	(91.3)
In sample re-testing, the person responsible for drawing the sample contacted to verify the validity of drawing the sample	25	(43.1)	51	(61.4)	102	(66.7)	55	(59.1)	77	(53.5)	< .001	310	(58.4)
In case of handling repeat critical values from the same patient, the usual practice is to:											< .001		
Report initial critical value and all subsequent critical values, regardless of previous results	25	(49.0)	36	(42.4)	59	(41.0)	36	(40.0)	68	(51.5)		224	(44.6)
Report worsening values, values that were "grossly different" from previous values, and values that moved in and out	5	(9.8)	29	(34.1)	23	(16.0)	27	(30.0)	37	(28.0)		121	(24.1)
Report repeated critical values once per interval of time	21	(41.2)	20	(23.5)	62	(43.1)	27	(30.0)	27	(20.5)		157	(31.3)
Reported critical laboratory values are documented:^†^													
In the computer system	15	(23.1)	31	(34.1)	56	(35.9)	38	(38.0)	45	(30.4)	.276	185	(33.0)
Written on the result form	8	(12.3)	19	(20.9)	24	(15.4)	12	(12.0)	21	(14.2)	.457	84	(15.0)
Documented in the laboratory register	14	(21.5)	23	(25.3)	45	(28.8)	19	(19.0)	30	(20.3)	.311	131	(23.4)
All of the above	39	(60.0)	43	(47.3)	73	(46.8)	48	(48.0)	84	(56.8)	.197	287	(51.2)
It is not documented	0	(0.0)	2	(2.2)	0	(0.0)	2	(2.0)	1	(0.7)	.205	5	(0.9)
No answers	6	(9.2)	1	(1.1)	4	(2.5)	0	(0.0)	3	(2.0)	.010	14	(2.5)
In case of documenting verbal communication on a log, the following is included:^†^													
Identification of patient	13	(20.0)	28	(30.8)	65	(41.7)	37	(37.0)	33	(22.3)	.001	176	(31.4)
Identification of sender	3	(4.6)	7	(7.7)	14	(9.0)	11	(11.0)	9	(6.1)	.521	44	(7.9)
Identification of recipient (person receiving the report)	6	(9.2)	19	(20.9)	31	(19.9)	23	(23.0)	22	(14.9)	.139	101	(18.0)
Critical test result reported	10	(15.4)	20	(22.0)	53	(34.0)	19	(19.0)	21	(14.2)	< .001	123	(22.0)
Date and time of reporting	11	(16.9)	22	(24.2)	51	(32.7)	25	(25.0)	31	(20.9)	.074	140	(25.0)
All information	31	(47.7)	47	(51.6)	55	(35.3)	47	(47.0)	99	(66.9)	< .001	279	(49.8)
No answers	16	(24.6)	6	(6.6)	25	(16.0)	7	(7.0)	5	(3.4)	< .001	59	(7.9)
The time from result availability to the responsible caregiver notification is measured	39	(63.9)	60	(69.0)	112	(75.7)	65	(69.1)	111	(76.0)	.025	387	(72.2)
Reasons for delay in reporting critical values:^†^													
Getting someone to accept the results	20	(30.8)	49	(53.8)	48	(30.8)	21	(21.0)	30	(20.3)	< .001	168	(30.0)
Reporting critical values to the physician responsible for the patient	15	(23.1)	46	(50.5)	52	(33.3)	16	(16.0)	23	(15.5)	< .001	152	(27.1)
Knowing the name of the assigned physician	7	(10.8)	29	(31.9)	19	(12.2)	12	(12.0)	30	(20.3)	.001	97	(17.3)
Provider contact information is not available	24	(36.9)	54	(59.3)	48	(30.8)	39	(39.0)	73	(49.3)	< .001	238	(42.5)
The person receiving the result is unwilling to read it back to ensure that it is correct	5	(7.7)	14	(15.4)	15	(9.6)	10	(10.0)	24	(16.2)	.294	68	(12.1)
List of critical values is too long	0	(0.0)	5	(5.5)	6	(3.8)	2	(2.0)	4	(2.7)	.450	17	(3.0)
Reporting critical results disrupts the workflow	0	(0.0)	19	(20.9)	8	(5.1)	6	(6.0)	12	(8.1)	< .001	45	(8.0)
Discharged patients at the time of reporting the result	11	(16.9)	41	(45.1)	49	(31.4)	35	(35.0)	51	(34.5)	.046	187	(33.4)
There are no difficulties	15	(23.1)	4	(4.4)	36	(23.1)	33	(33.0)	28	(18.9)	< .001	116	(20.7)
No answers	17	(26.2)	7	(7.7)	14	(9.0)	8	(8.0)	12	(8.1)	.001	58	(10.4)
There is a delay in reporting critical laboratory values	4	(7.0)	22	(25.9)	31	(20.8)	12	(12.6)	29	(21.0)	.112	98	(18.7)
Satisfied with the way a staff would report critical laboratory values	42	(73.7)	75	(86.2)	132	(86.8)	81	(84.4)	129	(93.5)	.002	459	(86.6)
The average time from result availability to the responsible caregiver notification in each of the following shifts													
Morning shift													
Range	0 – 600		10 – 4320		0 – 120		5 – 2880		0 – 1440			0 – 4320	
Mean ± SD	150.21 ± 760.53		153.13 ± 760.53		26.89 ± 28.66		303.54 ± 795.09		78.08 ± 250.62		.048	104.55 ± 408.16	
Median	120		10		30		60		30			30	
Evening shift													
Range	0 – 2880		10 – 60		0 – 120		5 – 120		0 – 1440			0 – 2880	
Mean ± SD	447.5 ± 765.82		19 ± 15.94		19.58 ± 27.08		61.25 ± 46.11		77.28 ± 250.43		< .001	94.44 ± 317.18	
Median	120		10		10		30		30			30	
Night shift													
Range	0 – 120		10 – 60		0 – 120		5 – 120		0 – 1440			0 – 1440	
Mean ± SD	83.21 ± 51.8		18.33 ± 14.99		21.94 ± 29.8		61.25 ± 46.11		77.28 ± 250.43		.389	60.36 ± 191.45	
Median	120		10		5		30		30			30	

n: Correct responses (%): Percentage SD: Standard deviation

^†^ Multiple responses are allowed

*p*: *p*-value (Statistically significant at *p* ≤ .05, highly significant at *p* ≤ .001)

### Commonly performed tests critical values

For some of the tests, there are differences in the ranges adopted by the hospitals. None of the participating individuals correctly identified the upper limits of the phosphorus, creatinine and prothrombin time (PT) tests. For the majority of the tests, differences in staff knowledge between the hospitals were statistically significant. The reported tests showed slight differences in critical values range between all hospitals. Table 4 lists the tests commonly performed in hospitals and their critical values according to hospital guidelines.

**Table 4:** Critical Values for Tests Commonly Performed at Participating Hospitals

	Hospital A				Hospital B				Hospital C				Hospital D				Hospital E				*p*	Total		
	V	N	n	%	V	N	n	%	V	N	n	%	V	N	n	%	V	N	n	%		N	n	%
Potassium																								
Lower Limit	2.5	1	0	0.0	2.5	27	25	92.6	2.8	15	1	6.7	2.5	4	0	0.0	2.8	37	34	91.9	< .001	84	60	71.0
Upper Limit	6.6	1	0	0.0	6.5	27	25	92.6	6.2	14	3	21.4	6.5	4	0	0.0	6.5	38	36	94.7	< .001	84	64	76.2
Unit	mmol/L	2	2	100	mmol/L	24	24	100	mmol/L	31	30	96.8	mmol/L	2	2	100	mmol/L	45	45	100	.566	104	103	99.0
Sodium																								
Lower Limit	120	1	1	100	120	27	25	92.6	120	14	11	78.6	120	4	0	0.0	120	38	34	89.5	.001	84	71	84.5
Upper Limit	160	1	1	100	160	27	25	92.6	160	14	11	78.6	160	4	0	0.0	160	38	36	94.7	< .001	84	73	86.9
Unit	mmol/L	2	2	100	mmol/L	23	23	100	mmol/L	31	23	74.2	mmol/L	2	2	100	mmol/L	45	45	100	.001	103	95	92.2
Magnesium																								
Lower Limit	0.4	1	0	0.0	0.4	25	24	96.0	0.5	12	11	91.7	0.4	2	0	0.0	0.5	36	36	100	< .001	76	71	93.4
Upper Limit	None	0	0	0.0	3.7	25	24	96.0	1.9	12	1	8.3	None	0	0	0.0	1.9	37	34	91.9	< .001	74	59	79.7
Unit	mmol/L	2	2	100	mmol/L	23	23	100	mmol/L	30	19	63.3	mmol/L	2	2	100	mmol/L	43	43	100	< .001	100	89	89.0
Calcium																								
Lower Limit	1.65	1	0	0.0	1.5	27	25	92.6	1.5	14	3	21.4	1.8	3	0	0.0	1.5	37	36	97.3	< .001	82	64	78.0
Upper Limit	3.25	1	1	100	3.5	27	24	88.9	3.2	15	1	6.7	3.5	2	0	0.0	3.2	37	36	97.3	< .001	82	62	75.6
Unit	mmol/L	1	1	100	mmol/L	24	24	100	mmol/L	29	18	62.1	mmol/L	2	2	100	mmol/L	43	43	100	< .001	99	88	88.9
Phosphorous																								
Lower Limit	0.32	1	1	100	0.36	23	22	95.7	None	0	0	0.0	0.3	0	0	0.0	None	0	0	0.0	> .999	24	23	95.8
Upper Limit	None	0	0	0.0	None	0	0	0.0	None	0	0	0.0	2.9	0	0	0.0	None	0	0	0.0		0	0	0.0
Unit	mmol/L	2	2	100	mmol/L	20	20	100	None	0	0	0.0	mmol/L	0	0	0.0	None	0	0	0.0		22	22	100
Bilirubin																								
Upper Limit	None	0	0	0.0	340	25	23	92.0	239	14	2	14.3	25	2	1	50.0	299	17	1	5.9	< .001	58	27	46.6
Unit	None	0	0	0.0	µmol/L	23	0	0.0	mmol/L	23	6	26.1	µmol/L	1	0	0.0	mmol/L	25	22	88.0	< .001	72	28	38.9
BUN																								
Upper Limit	None	0	0	0.0	R	0	0	0.0	None	0	0	0.0	None	0	0	0.0	28.56	28	19	67.9		28	19	67.9
Unit	None	0	0	0.0	R	0	0	0.0	None	0	0	0.0	None	0	0	100	mmol/L	28	26	92.9		28	26	92.9
Creatinine																								
Upper Limit	650	1	0	0.0	None	0	0	0.0	None	0	0	0.0	350	3	0	0.0	None	0	0	0.0		4	0	0.0
Unit	µmol/L	2	2	100	None	0	0	0.0	None	0	0	0.0	µmol/L	1	0	0.0	None	0	0	0.0	.333	3	2	66.7
PH																								
Lower Limit	None	0	0	0.0	R	0	0	0.0	7.2	7	0	0.0	None	0	0	25.0	7.2	35	29	82.9	< .001	42	29	69.0
Upper Limit	None	0	0	0.0	R	0	0	0.0	7.5	7	6	85.7	None	0	0	0.0	7.6	35	25	71.4	.654	42	31	73.8
PO2																								
Lower Limit	None	0	0	0.0	R	0	0	0.0	6.33	7	0	0.0	None	0	0	0.0	5.32	35	29	82.9	< .001	42	29	69.0
Unit	None	0	0	0.0	R	0	0	0.0	Kpa	15	6	40.0	None	0	0	0.0	Kpa	28	28	100	< .001	43	34	79.1
PCO2																								
Lower Limit	None	0	0	0.0	R	0	0	0.0	2.66	7	6	85.7	2.5	0	0	0.0	2.66	35	34	97.1	.309	42	40	95.2
Upper Limit	None	0	0	0.0	R	0	0	0.0	8.30	7	0	0.0	8.9	0	0	0.0	8.00	34	33	97.1	< .001	41	33	80.5
Unit	None	0	0	0.0	R	0	0	0.0	Kpa	15	6	40.0	Kpa	1	1	100	Kpa	32	32	100	< .001	48	39	81.3
Bicarbonate																								
Lower Limit	None	0	0	0.0	10	19	18	94.7	10	11	10	90.9	10	0	0	0.0	10	31	31	100	.242	61	59	96.7
Upper Limit	None	0	0	0.0	40	20	19	95.0	40	11	10	90.9	40	0	0	0.0	40	30	29	96.7	.755	61	58	95.1
Unit	None	0	0	0.0	mmol/L	19	19	100	mmol/L	19	17	89.5	mmol/L	1	1	100	mmol/L	29	26	89.7	.537	68	63	92.6
Neutrophil																								
Lower Limit	1	0	0	0.0	0.5	8	7	87.5	1	5	0	0.0	1	15	11	73.3	None	0	0	0.0	.004	28	18	64.3
Unit	10^9^/L	1	0	0.0	10^9^/L	8	7	87.5	10^9^/L	24	6	25.0	10^9^/L	11	10	90.0	None	0	0	0.0	< .001	44	23	52.3
Hemoglobin																								
Lower Limit	70	1	1	100	70	11	1	9.1	60	13	4	30.8	6.5	26	2	7.7	60	8	4	50.0	.010	59	12	20.3
Upper Limit	None	0	0	0.0	190	12	8	66.7	200	13	3	23.1	20	27	1	3.7	200	8	4	50.0	< .001	60	16	26.7
Unit	g/L	1	1	100	g/L	24	22	91.7	g/L	51	51	100	g/L	11	11	100	g/L	57	49	86	.033	144	134	93.1
Platelet																								
Lower Limit	50	1	1	100	20	3	1	33.3	30	13	0	0.0	20	26	7	26.9	20	14	12	85.7	< .001	57	21	36.8
Upper Limit	None	0	0	0.0	1000	9	0	0.0	1500	14	0	0.0	1000	25	19	76.0	1500	14	13	92.9	< .001	62	32	51.6
Unit (10^9^/L)	10^9^/L	1	0	0.0	10^9^/L	25	19	76.0	10^9^/L	44	14	31.8	10^12^/L	10	0	0.0	10^9^/L	54	0	0.0	< .001	124	33	26.6
aPTT																								
Upper Limit	100	0	0	0.0	120	0	0	0.0	70	7	0	0.0	120	20	8	40.0	70	3	0	0.0	.110	30	8	26.7
Unit	Sec	1	1	100	Sec	24	19	79.2	Sec	30	30	100	Sec	23	13	56.5	Sec	53	53	100	< .001	131	116	88.5
PT																								
Upper Limit	None	0	0	0.0	None	0	0	0.0	20	3	0	0.0	None	0	0	0.0	20	2	0	0.0		5	0	0.0
Unit	None	0	0	0.0	None	0	0	0.0	Sec	25	22	88.0	None	0	0	0.0	Sec	56	55	98.2	.085	81	77	95.1
INR																								
Upper Limit	5	0	0	0.0	6.0	1	0	0.0	None	0	0	0.0	4.5	22	9	40.9	3	1	1	100	> .999	24	9	37.5

V: Critical values as per hospital policy, N: Valid responses, n: Correct responses, %: Percentage of correct responses

*aPTT* Activated partial thromboplastin time, *PT* Prothrombin time, *INR* International normalisation ratio, *Sec *Seconds

*R* Retired (Critical value was removed from lab policy),* p*: *p*-value (statistically significant if *p* ≤ .05, highly significant if *p* ≤ .001)

### Comparing the three outcomes based on socio-demographic groups

Regarding the upper and lower limits of critical values and their units, the hospitals showed highly significant statistical differences, but respondents from hospitals B and E gave the most correct answers. Satisfaction with the way staff report critical values was subject to significant difference between hospitals. Individuals from haematology and biochemistry units gave the most correct critical values and units, and differences between the units were highly significant differences (*p* < 0.001). The data revealed a statistically significant difference (*p* = 0.015) between gender in the perception of delays in the reporting of critical laboratory values. There is a statistically significant difference associated with age group regarding correct answers of limits and units for critical values. In addition, highly statistically significant differences for all scenarios were observed for nationality, and different professions also plays a role in total. In addition, the different types of doctor showed statistically significant differences in laboratory critical values when reporting is delayed whereas technicians in regards to the responses showed satisfaction with the way critical values are reported. Furthermore, positions did not show any significant difference in regards to limits and units, whereas delays and satisfaction in reporting were subject to statistically significant differences (*p* = 0.049 and 0.001, respectively). Finally, views on the delay in critical value reporting when compared to limits and units, showed a statistically significant difference. A highly significant statistical difference was found between satisfaction in reporting critical laboratory values and perception of delays. A statistically significant difference was found between satisfaction with reporting procedures and the limits and units assessment. Table 5 shows a comparison between socio-demographic groups in regards to three scenarios.

**Table 5:** Comparison between socio-demographic groups in regards to three outcomes

	Correct answers of upper and lower limits and units of critical values			There is a delay in reporting critical laboratory values				Satisfied with the way a staff would report critical laboratory values			
	Median	(IQR)	*p*	Yes	No	I don’t know	*p*	Yes	No	I don’t know	*p*
Hospital			< .001				.112				.002
A	0.0	(0)		4	49	4		42	8	7	
B	2.0	(12)		22	57	6		75	4	8	
C	0.0	(3)		31	106	12		132	16	4	
D	0.0	(1)		12	76	7		81	9	6	
E	3.0	(12.75)		29	103	6		129	8	1	
Unit			< .001				.090				< .001
Microbiology	0.0	(0)		22	77	9		96	9	5	
Hematology	3.0	(4)		38	124	6		155	11	3	
Biochemistry	7.0	(18)		19	91	8		107	9	3	
Histopathology	0.0	(0)		10	29	7		29	8	10	
Immunology	0.0	(0)		1	22	2		18	4	3	
Virology	0.0	(0)		6	31	0		33	3	1	
Molecular genetics	0.0	(0)		0	5	0		5	0	0	
Reception	0.0	(0)		1	5	1		6	0	1	
Gender			.094				0.015				.462
Male	0.0	(3)		27	162	9		177	16	7	
Female	0.0	(5)		70	229	26		281	29	19	
Age			.006				.085				.123
Below 30 years	0.0	(3)		41	113	12		151	9	8	
30–45 years	0.0	(3)		43	209	21		227	31	17	
46–55 years	1.0	(4)		10	36	2		44	4	1	
Over 55 years	3.0	(6)		4	32	0		36	1	0	
Nationality			< .001				< .001				< .001
Kuwaiti	0.0	(5)		43	121	23		142	30	17	
Non-Kuwaiti	0.0	(2)		55	269	11		315	15	9	
Profession			< .001				.030				< .001
*Doctor*	0.0	(1)	.099	6	46	8	.002	40	12	8	.730
Assistant register	0.0	(0)		2	2	3		3	2	2	
Register	0.0	(1)		0	21	3		17	4	3	
Senior register	0.0	(4)		1	12	2		11	3	1	
Specialist	0.0	(2)		0	7	0		3	2	2	
Senior specialist	0.0	(0)		0	3	0		3	0	0	
Consultant	2.0	(7)		3	1	0		3	1	0	
*Technician*	0.0	(4)	.278	92	343	27	.475	417	33	18	.039
Assistant practitioner	0.0	(11.25)		2	24	2		27	0	1	
Practitioner	0.0	(2)		4	27	3		29	3	2	
Senior practitioner	0.0	(3)		4	13	2		14	4	2	
Assistant technician	0.0	(3.75)		9	12	1		19	3	0	
Technician	0.0	(4)		50	180	13		228	12	7	
Senior technician	3.0	(7)		8	27	2		30	6	1	
Specialist	0.0	(3)		7	26	1		31	2	2	
Senior specialist	0.5	(3)		7	34	3		38	3	3	
Position			.116				.049				< .001
Head of department	0.0	(6)		1	4	0		3	2	0	
Head of unit	0.0	(2)		2	6	0		6	1	1	
Laboratory doctor	0.0	(1)		3	37	8		32	9	7	
Head of laboratory technicians	0.0	(2)		5	9	1		10	3	2	
Technician	0.0	(4)		86	320	26		395	26	16	
There is a delay in reporting critical laboratory values			.049								< .001
Yes	0.0	(4)						75	17	6	
No	0.0	(4)						362	21	8	
I don’t know	0.0	(4)						17	6	12	
Satisfied with the way a staff would report critical laboratory values			.001								
Yes	0.0	(4)									
No	0.0	(2)									
I don’t know	0.0	(0)									

*IQR* Interquartile range, *p*: *p*-value (statistically significant if *p* ≤ .05, highly significant if *p* ≤ .001)

## Discussion

Measuring and reporting critical values in the laboratory plays a large clinical role in patient safety and well-being [10]. On that basis, timeframes for the reporting of critical values should be set by each laboratory in consultation with clinicians whether laboratory doctors or other departments physicians [11]. In our study, we estimate the average number of critical value notifications in a one-year period is between 126,000 and 3,600,000, according to studies from China and India [12, 13]. The current study showed that approximately 88% of staff participating considered critical value notification as an essential procedure to practice when an alarming value arises. Many schemes for the accreditation of medical laboratory policies and practices consider the communication of critical values as essential [14], and our respondents seem to agree. Notification is usually ineffective when contacting the responsible physician by telephone [15]. Telephone notification has advantages according to AlSadah et al. [8], such as affording the opportunity to read back the results with immediate explanation, if needed, to minimise errors. Our study revealed that two-thirds of our responses declared using the telephone for critical values reporting, further studies should be done to ensure the control of reporting time and reducing communication errors. Nonetheless, wireless technologies nowadays play a large role in many ways, including critical value notification and reporting, but only around one-third of responses to our survey indicated the use of these technologies to deliver the notification which is not matching subsequent study. A study in India [16] suggested that increased usage of wireless technology in laboratories will improve quality metrics including turnaround time.

To ensure good critical value reporting, the existence of a written procedure for reporting and a comprehensive list of critical values should aid communication [17]. Around 90% of respondents claimed to know of the former, which is a good indication that good reporting is practiced. Before reporting any critical value, the sample should be checked for potential quality issues, for example, by serum or anticoagulated plasma clotting time [18]. Around 87% responses indicated knowledge that a comprehensive list existed, which might acknowledge the importance of critical value notification. Reporting critical values according to a written protocol will address quality assurance and avoid tests reference ranges errors [16]. Medical societies—usually guided by medical professionals representing a particular specialised field—play a role in keeping their community informed about the latest research and developments in that field; following their recommendations aims to ensure that patients receive the best treatment available [19]. According to our results, 32.1% of surveyed staff indicated that medical society recommendations were followed for developing critical value lists in their setting. Consistent with an earlier study, low percentages of responses indicated that critical value lists are based on published literature (21%) and clinician’s opinions (23%). Our prior results showed low knowledge about the policies and guidelines relating to the practice of critical value notification. Having a policy for assessing the timeliness (time frame requires fast response) of critical value reporting is crucial for improvement of patient care [20], and around 84% of respondents indicated their setting had such a policy in place. Notification time has been described by Lippi and Mattiuzzi as an indicator of the quality of patient safety [14]. A study by Piva et al. [21] stated that both technicians and laboratory physicians are responsible for the notification of critical values, whereas in the hospitals we surveyed, technicians are the members of staff who perform the notification of any critical value by around 63% of responses. Usually, the physician who ordered the tests will receive the critical value notification, If they are unavailable, a nurse will receive it, and finally, if necessary, any physician on call [8]. Our findings mirrored this, showing that the physician ordering the test (63%), nurses (60%) and any physician on call (41%) receive the notification. According to a study by Clavijo et al. [22], the hierarchical notification system (with nurses excluded) is time dependent, which means there is a specified time in which to notify the responsible physician. If they cannot be reached, the next in charge should be notified. Widespread knowledge about critical value communication upon identification made a significant difference with notification practices in this study, approximately 92% of participating hospital staff stated they notify immediately. Each laboratory has their own policy for reporting critical values [23]. Approximately 45% of survey participants indicated that all critical values from a patient, including repeat measurements, are reported, which roughly matches the current study. The documenting of critical value reporting is required by the joint commission and other institutes [24]. All documentation is done for the purposes of quality monitoring, including notification time measurements, as reported by Piva et al. [21]. According to former study, approximately half of responses indicated the use of three different documentation methods. An intervention study by Bhartia et al. [25] aimed at improving the quality of critical value reporting recommended, sharing delayed reports with responsible caregiver to avoid future obstacles between technologists and physicians. Furthermore, prior studies reported delays in reporting critical values; thus, solving the problem of delayed reporting will improve the quality of the service. Less than 19% of responses to our survey confirmed a delay in reporting; this is considerable, and lowering it will help improve service quality, as prior studies have asserted. All previous studies showed and assessed the knowledge of different practices towards critical value notification policies and guidelines to our study.

Genzen and Tormey [26] observed that notification of critical values should be done by a technician on the team performing the measurement, to ensure compliance with the joint commission standards which is a non-profit organisation accredits more than 22,000 healthcare organisations between hospitals and primary clinics in the United States of America. Around 92% of responses indicated that laboratory staff are trained in reporting critical values, that is, the staff are familiar with the critical value limits and how to notify when these are breached [27]. According to Ye et al. [28], training programmes to improve delivery of critical values between relevant personnel should be undertaken. Our study found that delays in critical value reporting were significantly different between physicians and technicians, which might be due to policies and the guidelines not being followed. Past studies co-ordinated our current results and showed the attitude towards the policies and guidelines regarding critical value notification. Managing the notification time and following a flowchart to report critical values can reduce reporting errors, improve notification of critical values and further guarantee patient safety [15]. Dixon et al. [29] stated that result notification should be available in health departments within 1440 to 2880 min. Approximately 88% of respondents confirmed their setting has a policy for dealing with repeated measurement of critical values. Typically, repeating a critical value measurement does not affect the result [30]. Nonetheless, this study did not explore if there is a specific list of tests to be repeated in case a critical value arises. Furthermore, Saffar et al. [24] argue that repeating a test is unnecessary and might actually affect patient safety through delays in taking clinical action and the waste of resources [31], which is not similar to our results. A read-back policy for critical value notification is often required of the responsible person delivering it [32], and nearly 79% of respondents assured they have such a policy to follow. In addition, it is important to practice the read-back guidelines when reporting critical values to avoid communication errors; it is also a requirement of accreditation programmes [33].

Each laboratory should have their own list of critical values and recommendations taken from the medical societies, as Arbiol-Roca and Dot-Bach [34] suggest. After setting lists of critical values, their own results can be compared against those limits to meet the needs of patients and clinicians, to avoid outliers and to ensure nationwide standards [5, 35]. Furthermore, the use of unique ranges for distinct populations grouped by age and diagnosis is needed to avoid inappropriate assignment as a critical value. Valiathan et al. [36] highlighted that many studies report that age and diagnosis can affect reference ranges for lymphocytes, so checking the patient's age and diagnosis before reporting or notifying of any critical value is recommended. Our responses, compared to prior studies, showed unique ranges for age and diagnosis (63% and 41%, respectively), showing staff moderately following that concept. A separate study advised each laboratory to set their critical values list according to clinical needs [10]. Our findings revealed some differences between hospitals in the limits set for critical values, which contradicts the findings of a previous study. Such differences affect patient safety and the quality of the services provided from governmental hospitals.

Finally, because the Ministry of Health in Kuwait is responsible for almost every aspect of its hospitals, a clear notification system showing exact notification time limits is recommended for improving the quality of patient services. In addition, it is recommended that all laboratories clarify their policies concerning, for example, read-back and repeat testing after critical values have been notified. Furthermore, a unified list of upper and lower critical value limits is advisable to avoid confusion between hospitals over results.

### Strengths and limitations

This study has some limitations to acknowledge. One of these is missing responses owing to respondents skipping questions. Each part of the questionnaire is considered a separate and independent section. To control that, one answered question from the questionnaire is considered a valid response and included in the analysis. The number of participating laboratories is somewhat small, a reflection of the government sector, and can be regarded as a limitation. Only tertiary hospitals were included, whereas primary care and private laboratories were excluded from this study. Furthermore, the entry and cleaning of data acquired using written questionnaires (hard copies) is laborious, although a respondent skipping questions leads to missing responses, which is considered a limitation. Also, the tool assesses staff attitudes and perceptions but not knowledge (items in Table 4 are the exceptions to this). The strength of the study is that its findings can be generalised across laboratories in Kuwait due to the use of total population sampling. This is a purposive sampling technique, a type of non-probability sampling that allows the analytical generalisation about a studied population.

## Conclusions

Based on the responses of the participating hospitals, the policies and procedures for notifying on critical values at those settings are not clear. A range of responses highlighted failures to implement policy or procedures, and some staff even thought it is not essential to report if a critical value was notified. Unified critical value policies should be distributed among participating laboratories to avoid variation in reporting practices. The availability of clear policies or guidelines instructing staff on how and when to report a critical value was significantly different between hospitals. Unavailability of such guidelines leads to notifications being delayed or miscommunications owing to a lack of patient information or missed documentation. Policies and guidelines should be clear and implemented by the Ministry of Health to avoid confusion over their mandate, rather than simply being recommendations without follow-up. Notification of critical values showed some disparities between laboratories in terms of time limits and the method of delivering the notification. As our study found that differences in critical value limits between hospitals were statistically significant, that might affect a patient’s safety in the long term. Creating lists of critical values, showing the limits and the maximum notification times, and disseminating it to all laboratories, might reduce critical cases in patients and increase the quality of medical services. In the long term, the results of this study are expected to steer government policy for improving laboratory practices and patient-centred policies.

## Supplementary Information

**Additional file 1: Appendix 1.** Laboratory Critical Values Survey (Arabic version). **Appendix 2.** Laboratory critical values questionnaire (English version).

## Data Availability

The datasets generated and/or analysed during the current study are not publicly available owing to Ministry of Health restrictions. However, datasets are available from the corresponding author upon reasonable request.

## Abbreviations

ANOVA: Analysis of Variance
aPTT: Activated partial thromboplastin time
BUN: Blood urea nitrogen
IQR: Interquartile range
n: Correct responses
N: Valid responses
pCO_2_: Partial pressure of carbon dioxide
pO2: Partial pressure of oxygen
PT: Prothrombin time
INR: International normalisation ratio
Sec: Seconds
R: Retired (critical value was removed from laboratory policy)
V: Critical values as per hospital policy
MOH: Ministry of Health
