# Supplementary material for: Baseline assessment of staff perception of critical value practices in government hospitals in Kuwait
Source: BMC Health Serv Res. 2022 Aug 3;22:986. doi: 10.1186/s12913-022-08329-z (PMC9347105; doi:10.1186/s12913-022-08329-z)
Supplement: Supplementary file 1 — Additional file 1: Appendix 1. Laboratory Critical Values Survey (Arabic version). Appendix 2. Laboratory critical values questionnaire (English version). [file 12913_2022_8329_MOESM1_ESM.docx]

Appendix 1

**استبيان القيم المعملية الحرجة**

**Laboratory Critical Values Survey**

**القيم المعملية الحرجة** هي النتائج التي تقع خارج المعدلات الطبيعية إلى درجة أنه قد تشير إلى وجود حالة تهدد حياة المريض إذا لم يتم التدخل السريع.

**البيانات الديمغرافية**

1. **اسم المستشفى**
2. **الوحدة :**

- الميكروبيولوجي(Microbiology)
- الهيماتولوجي(Hematology)
- البيوكمستري(Biochemistry)
- الستوباثولوجي (HistoPathology)
- امينولوجي (Immunology)
- الفيرولوجي(Virology)
- .أخرى, رجاء التحديد:.......................

1. **المسمى الوظيفي:**

**طبيب:**

- مساعد مسجل
- مسجل
- مسجل أول
- اختصاصي
- اختصاصي أول
- استشاري

**فني:**

- ممارس مبتدئ
- ممارس
- ممارس أول
- فني مبتدئ
- فني
- فني أول
- اختصاصي
- اختصاصي أول

**4. المنصب الوظيفي:**

- رئيس قسم
- رئيس وحدة
- طبيب مختبر
- رئيس اختصاصي
- فني

1. **الجنس**:

- ذكر
- أنثى

1. **العمر:**

- أقل من 30
- 30-45 عاما
- 46-55 عاما
- أكثر من 55 عاما

1. ا**لجنسية**

- كويتي
- غير كويتي

1. **السياسات والإجراءات**
2. في هذا المختبر، نعتبر إبلاغ القيم الحرجة

- إجراء غير هام
- إجراء مهم إلى حد ما
- إجراء ضروري

1. يوجد لدينا إجراءات مكتوبة لتحديد القيم المعملية الحرجة و كيفية الإبلاغ عنها

- نعم
- لا
- لا أعرف

1. هناك قائمة شاملة من القيم الحرجة (لأمراض الدم عامة، تجلط الدم والتحاليل الكيميائية)

- نعم
- لا
- لا أعرف

1. إذا كان هناك قائمة شاملة من القيم الحرجة، فعدد التحاليل التي تشتمل عليها، ................... تحليل
2. صياغة قائمة القيم الهامة، إن وجدت، اعتمدت علي

- بيانات من الدوريات العلمية
- آراء الأطباء في مؤسساتهم
- توصيات الجمعيات الطبية
- توصيات الشركات الصانعة
- مراجعة الممارسة المختبرية
- استعراض الأقران المخبرية غير الرسمية
- أخري .......................

1. هل يتم تدريب العاملين في المعمل على عمليه إبلاغ القيم المعملية الحرجة؟

- نعم
- لا
- لا أعرف

1. هل يتم تحديث إجراءات تحديد القيمة المعملية الحرجة وكيفية إبلاغها بشكل دوري؟

- نعم
- لا
- لا أعرف

1. في هذا المعمل، هل تختلف قيم النتائج المعملية الحرجة باختلاف عمر المريض؟

- نعم
- لا
- لا أعرف

1. في هذا المعمل، هل تختلف قيم النتائج المعملية الحرجة باختلاف التشخيص؟

(e.g., repeated critical factor VIII activity levels were not reported for patients with known severe hemophilia A; repeated critical levels for patients with cancer were reported every 7 days).

- نعم
- لا
- لا أعرف

1. هل توجد سياسة لتقييم دقة توقيت إبلاغ النتائج المعملية الحرجة؟

- نعم
- لا
- لا أعرف

1. هل لديكم سياسة بشأن كيفية إدارة تكرار القيم المعملية الحرجة (عينه لاحقه لفحص معين على نفس المريض).؟
   - نعم
   - لا
   - لا أعرف
2. في حالة إبلاغ النتائج المعملية الحرجة شفهيا أو عن طريق التليفون، هل تتضمن سياسة المعمل الإعادة الشفهية للنتيجة من قبل الشخص المتلقي للنتيجة للتأكد من صحة سماعها؟

- نعم
- لا
- لا أعرف

1. **عملية الإبلاغ عن القيم المعملية الحرجة**
2. في هذا المعمل، هل يتم إبلاغ القيم الحرجة فور التعرف عليهم من قبل موظفي المعمل؟

- نعم
- لا
- لا أعرف

1. عند وجود قيمة حرجة لأحدي المرضي، من هو الشخص المسئول عن إبلاغ تلك القيمة لمقدمي الرعاية الصحية للمريض؟ (الرجاء اختيار كل ما ينطبق)

- أخصائي التحاليل / النائب الموجود بالمعمل
- فني المعمل
- أخري (يرجي التحديد) ........................

1. من هو الشخص المسئول عن تلقي بلاغات المعمل للقيم الحرجة (الرجاء اختيار كل ما ينطبق)؟

- الطبيب الذي أوصي بعمل الاختبار
- الممرضة الموجودة بجناح المرضي
- أي طبيب تحت الاستدعاء on call
- أي شخص يعمل بجناح المرضي
- أخرى (يرجى التحديد) ...............

1. يتم الإبلاغ عن القيم الحرجة من المعمل إلى مقدمي الرعاية بشكل رئيسي عن طريق (الرجاء اختيار كل ما ينطبق)

- إرسال التقرير لجناح المرضي
- الهاتف
- الحاسوب
- الاتصال المباشر للالتقاء والتحدث إلى الطبيب المسئول عن المريض
- فاكس
- جميع ما سبق ذكره

1. هل يتم استخدام أي تقنيات لاسلكية لإبلاغ تقرير القيم الحرجة؟

- نعم
- لا
- لا أعرف

1. هل يتم إعادة اختبار العينة للتأكد من النتائج المعملية الحرجة؟

- نعم
- لا
- لا أعرف

1. إذا تم إعادة اختبار العينة، هل يتم الاتصال بالشخص المسئول عن سحب العينة للتأكد من صحه عمليه السحب

- نعم
- لا
- لا أعرف

1. في حالة التعامل مع القيم الحرجة المتكررة من نفس المريض، فإن الممارسة المعتادة هي:

- إبلاغ القيمة الحرجة الأولية وجميع القيم الحرجة اللاحقة، بغض النظر عن النتائج السابقة
- إبلاغ القيم التي تزداد سوءا، والقيم التي كانت "مختلفة بشكل كبير" من القيم السابقة، والقيم التي انتقلت داخل وخارج النطاق الحرج
- الإبلاغ عن القيم الحرجة المتكررة بعد مضي وقت محدد (على سبيل المثال، القيم الحرجة التي تحدث بعد أكثر من 12 ساعة بعد إبلاغ آخر قيمه حرجه)

1. **تسجيل عملية إبلاغ القيم المعملية الحرجة بسجلات المعمل**
2. كيف يتم توثيق القيم المعملية الحرجة التي يتم الإبلاغ عنها؟ (الرجاء اختيار كل ما ينطبق)

- التسجيل في الكمبيوتر
- تسجل على نتيجة تحليل المريض
- تدون في سجلات المعمل
- كل ما سبق
- لا يتم توثيقها

1. في حالة توثيق التواصل الشفهي على سجل المعمل، يتم تضمين البيانات التالية (الرجاء اختيار كل ما ينطبق)

- تعريف المريض (الأول واسم العائلة، المعرف الفريد)
- تعريف المرسل (تقني إصدار التقرير اللفظي)
- تعريف المستقبل (الشخص الذي يتلقى التقرير)
- نتيجة الاختبار المعملي الحرج الذي يتم الإبلاغ عنه
- تاريخ ووقت الإبلاغ عن النتيجة

1. هل يتم قياس الوقت منذ توافر النتيجة إلى الوقت الذي يتم إبلاغ الطبيب المسئول؟

- نعم
- لا
- لا أعرف

1. ما هو متوسط ​​الوقت على توصيل القيم الحرجة لمريض في القسم الداخلي بدءا من الوقت الذي كانت النتيجة متاحة في كلا من الورديات التالية:

- ورديه صباحيه (morning shift) ................................................
- ورديه مسائية (ُEvening shift) .................................................
- ورديه ليليه (Night shift) ........................................................

1. **32.يرجى الإشارة إلى القيم الحرجة العالية والمنخفضة للفحوصات المخبرية التالية:**

| **Test** | **Lower limits** | **Upper limits** | **Unit** |
| --- | --- | --- | --- |
| Potassium |  |  |  |
| Sodium |  |  |  |
| Magnesium |  |  |  |
| Calcium |  |  |  |
| Neutrophil |  |  |  |
| Hemoglobin |  |  |  |
| Platelet |  |  |  |
| Prothrombin time (PT) |  |  |  |
| INR |  |  |  |
| Activated partial thromboplastin time (aPTT); |  |  |  |
| PH |  |  |  |
| PO_2_ |  |  |  |
| PCO_2_ |  |  |  |
| Chloride |  |  |  |
| Phosphorus |  |  |  |
| Bilirubin |  |  |  |
| Bicarbonate |  |  |  |
| BUN |  |  |  |
| Creatinine |  |  |  |

1. **.33إذا كان هناك تأخير في الإبلاغ عن القيم الحرجة، ما سبب ذلك؟ (الرجاء اختيار كل ما ينطبق)**

- الحصول على شخص لقبول النتيجة
- إبلاغ القيم الحرجة إلى الطبيب المسئول عن المريض
- معرفة اسم الطبيب المسئول
- البيانات اللازمة للتواصل مع الطبيب المسئول غير متوافره
- الشخص المتلقي للنتيجة غير راغب في إعادتها شفهيا للتأكد من صحة سماعها
- قائمه النتائج الحرجة طويله جدا
- إبلاغ النتائج الحرجة يعطل سير العمل
- خروج المريض عند وقت إبلاغ النتيجة
- لا يوجد صعوبات

1. **الرضا عن عمليه الإبلاغ عن النتائج المعملية الحرجة**
2. هل أنت راض عن طريقة الإبلاغ عن للقيم المعملية الحرجة؟
   - نعم
   - لا
   - لا أعرف
3. هل تعتقد أن هناك تأخير في الإبلاغ عن القيم المعملية الحرجة؟
   - نعم
   - لا
   - لا أعرف

Appendix 2

**Laboratory critical values questionnaire**

Critical values refer to values or interpretations that, if left untreated, could be life threatening or place the patient at serious risk.

**Demographic Data**

1. **Hospital name**
2. **Unit name**

- Microbiology
- Hematology
- Biochemistry
- Histopathology
- Immunology
- Virology

1. **Job Title**

- **Doctor**
- Assistant registrar
- Register
- Senior register
- Specialist
- Senior Specialist
- Consultant
- **Technician**
- Assistant Practitioner
- Practitioner
- Senior practitioner
- Assistant technician
- Technician
- Senior technician
- Specialist
- Senior Specialist

1. **position**

- Head of Department
- Head of Unit
- Laboratory Doctor
- Head of laboratory technicians
- Technician

1. **Gender**

- Male
- Female

1. **Age**

- Less than 30 years
- 30 – 45 years
- 46 – 55 years
- Over 55 years

1. **Nationality**

- Kuwaiti
- Non-Kuwaiti

1. **policies and procedures**
2. **In this laboratory, we consider critical values reporting:**

- Minor Policy
- Somewhat Important
- An essential Procedures

**9. Do you have written procedures to determine critical laboratory values and how to report them?**

- Yes
- No
- I don't know

1. **Do you have a comprehensive list of critical values?**

- Yes
- No
- I don't know

1. **If there is a comprehensive list of critical values, number of tests in the list is ………….test**
2. **Critical values list was developed Bases upon:**

- Published literature
- Opinion of clinicians
- Medical societies' recommendation
- Manufacturer's recommendations
- Review of laboratory practice
- Informal laboratory peer review
- Others; please specify ………….

1. **Are laboratory personnel trained in the process of reporting critical laboratory values?**

- Yes
- No
- I don't know

1. **Are Procedures for determining critical laboratory values and how to report them regularly updated**

- Yes
- No
- I don't know

1. **In this laboratory; do you have unique ranges for distinct population by age?**

- Yes
- No
- I don't know

1. **In this laboratory; do you have unique ranges for distinct population by diagnosis?**

- Yes
- No
- I don't know

1. **Do you have policy for assessing timeliness of reporting?**

- Yes
- No
- I don't know

1. **Do you have policy on how to manage the repetition of critical laboratory values (sample later for a specific examination on the same patient)?**

- Yes
- No
- I don't know

1. **If critical laboratory results are communicated verbally or by telephone, does the laboratory policy require read-back of the result by the person receiving the result to ensure that it is correct?**

- Yes
- No
- I don't know

1. **Process of critical value reporting**
2. **In this laboratory, are critical values communicated immediately upon identification by laboratory personnel?**

- Yes
- No
- I don't know

1. **When there is a critical value for one of the patients, who is responsible for communicating that value to the patient's health care providers? (Please select all that apply)**

- Senior staff
- Laboratory technician
- Others; please specify ………….

1. **Who is responsible for receiving laboratory notifications for critical values? (please select all that apply)**

- Physicians ordering the test
- Nurses
- Any physician on call
- Any people working on the ward
- Others; please specify ………………

1. **Critical values are reported from the laboratory to the caregiver mainly by (please select all that apply)**

- Sending test report to ward
- Telephone
- Computer
- Direct contact with requesting physician
- Fax
- All tools

1. **Are any wireless technologies used to report critical values?**

- Yes
- No
- I don't know

1. **Is the sample re-tested to confirm critical laboratory results?**

- Yes
- No
- I don't know

1. **If the sample has been re-tested, is the person responsible for drawing the sample contacted to verify the validity of drawing the sample?**

- Yes
- No
- I don't know

1. **In case of handling repeated critical values from the same patient, the usual practice is to:**

- Report initial critical value and all subsequent critical values, regardless of previous results
- Report worsening values, values that were “grossly different” from previous values, and values that moved in and out of the critical range.
- Report repeated critical values once per interval of time

1. **Critical values documentation and monitoring**
2. **How are reported critical laboratory values documented? (Please select all that apply)**

- In the computer system
- Written on the result form
- Documented in the laboratory register
- All of the above
- It is not documented

1. **In case of documenting verbal communication on a log, the following is included ((Please select all that apply)**

- Identification of patient
- Identification of sender
- Identification of recipient (person receiving the report
- Critical test result reported
- Date and time of reporting

1. **Is the time taken from result availability to the time that the responsible caregiver is notified measured?**

- Yes
- No
- I don't know

1. **What is the average time from result availability to the time that the responsible caregiver is notified available in each of the following shifts?**

- Morning shift................................................
- Evening shift .................................................
- Night shift........................................................

1. **IV: Please indicate the high and low critical values for the following laboratory tests:**

| **Test** | **Lower limits** | **Upper limits** | **Unit** |
| --- | --- | --- | --- |
| Potassium |  |  |  |
| Sodium |  |  |  |
| Magnesium |  |  |  |
| Calcium |  |  |  |
| Neutrophil |  |  |  |
| Hemoglobin |  |  |  |
| Platelet |  |  |  |
| Prothrombin time (PT) |  |  |  |
| INR |  |  |  |
| Activated partial thromboplastin time (aPTT); |  |  |  |
| pH |  |  |  |
| pO_2_ |  |  |  |
| pCO_2_ |  |  |  |
| Chloride |  |  |  |
| Phosphorus |  |  |  |
| Bilirubin |  |  |  |
| Bicarbonate |  |  |  |
| BUN |  |  |  |
| Creatinine |  |  |  |

1. **V: If there is a delay in reporting critical values, why? (Please select all that apply)**

- Getting someone to accept the result
- Reporting critical values to the physician responsible for the patient
- Knowing the name of the assigned physician
- provider contact information is not available
- The person receiving the result is unwilling to read it back to ensure that it is correct
- list of critical values is too long
- Reporting critical results disrupts the workflow
- Discharged patients at the time of reporting the result
- There are no difficulties

**VI. Satisfaction with the process of reporting critical laboratory results**

1. **Are you satisfied with the way you report critical laboratory values?**

- Yes
- No
- I don't know

1. **Do you think there is a delay in reporting critical laboratory values?**

- Yes
- No
- I don't know
